# Spontaneous pneumomediastinum presenting as rhinolalia and chest pain

**DOI:** 10.1308/003588412X13373405387339

**Published:** 2012-05

**Authors:** RWF Breakey, G Walker, W Oldfield

**Affiliations:** Imperial College Healthcare NHS Trust,UK

**Keywords:** Pneumomediastinum, Management, Cardiothoracic

## Abstract

This report discusses a case of spontaneous pneumomediastinum in a 25-year-old medical student. The patient presented with chest pain and a tonal change in voice. Symptoms occurred after an episode of stretching and were exacerbated by coughing. There was no history of underlying respiratory disease and he was a non-smoker. Management was conservative. At the four-week follow-up appointment, bronchoscopy and computed tomography of the thorax demonstrated complete resolution. Spontaneous pneumomediastinum is uncommon, with rhinolalia being a rare presenting feature. It should be considered as a differential diagnosis in patients with chest pain associated with a change in voice. A detailed history may reveal preceding activities associated with raised intrathoracic pressure.

## Case history

A 25-year-old medical student presented to the accident and emergency department ten hours after developing a sudden onset, burning, retrosternal chest pain that radiated to the neck. This was associated with a change in the sound of his voice. The pain evolved after stretching while studying in the medical library. In an attempt to clear his throat, the patient had coughed forcefully after the pain had developed. There was no history of trauma, vomiting or recent strenuous exertion. The pain was made worse by swallowing, coughing and lying flat. He had no previous respiratory illness and was a non-smoker.

On examination, the patient was found to have a high pitched, nasal voice. He was haemodynamically stable with a heart rate of 79bpm and oxygen saturation of 99% on room air. Inspection revealed a swollen neck, with post auricular, cervical, supraclavicular and subclavicular tactile crepitations suggestive of subcutaneous emphysema. This extended to the patient’s forearms. On auscultation, crepitations were heard over the mediastinum in time with both diastolic and systolic components of the heartbeat.

A chest radiograph revealed considerable subcutaneous emphysema with air present in the mediastinum and extending into the pericardium. Computed tomography (CT) of the neck, thorax and abdomen confirmed pneumomediastinum with air tracking into the cervical fascia to the skull base.

The patient was managed conservatively with simple analgesia and discharged the same day. Symptoms soon resolved and the patient reports no further complications. Follow-up broncoscopy and CT four weeks later revealed complete resolution. No endobronchial lesions were identified and no underlying cause was identified.

## Discussion

Spontaneous pneumomediastinum is a relatively rare condition accounting for around 1 in 30,000 emergency department attendances annually.^1^ It most commonly affects young, tall, thin men.^2^

The first description of spontaneous pneumomediastinum in the literature appears to be in 1617 when Louise Bourgeois, the midwife to the queen of France, documented swelling of the neck and chest pain in relation to increased intrathoracic pressure occurring during parturition.^3^ More recently, Hamman depicted spontaneous pneumomediastinum in 1939, when he first recorded his finding of crackles, bubbles or churning sounds heard with each contraction of the heart.^4^ These characteristic findings have become known as Hamman’s sign and are found with varying frequency in cases of spontaneous pneumomediastinum.^5^

The pathophysiology behind spontaneous pneumomediastinum is based on increased intrathoracic pressure such as that produced by exaggerated Valsalva manoeuvres, cough, emesis or parturition, leading to alveolar rupture and dissection of air along vascular shafts and connective tissue planes towards the mediastinum.^6^ In many cases, this air ascends the communicating cervical spaces, producing subcutaneous cervical emphysema.^7^ Our case illustrates two of these causes. The pneumomediastinum occurred while stretching with breath held and was made worse when the patient coughed repeatedly in an attempt to clear his throat.

**Figure 1:**
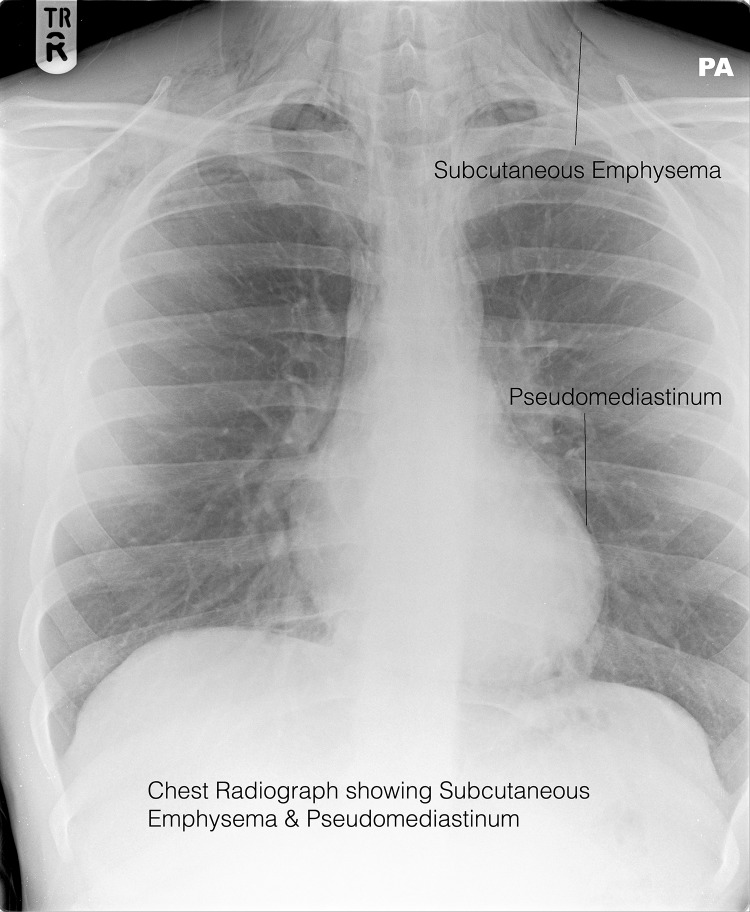
Chest radiograph demonstrating pneumomediastinum and extensive cervical subcutaneous emphysema

**Figure 2:**
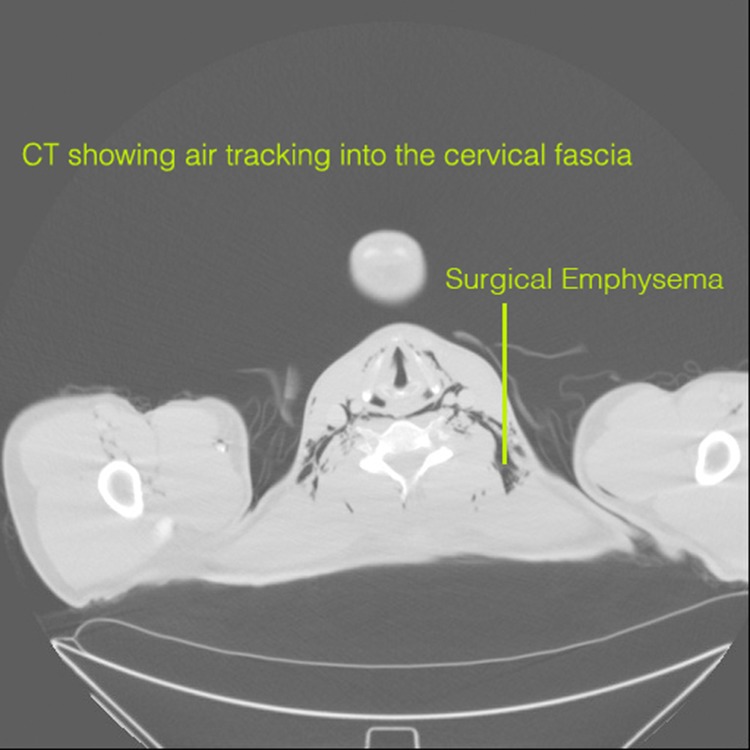
Computed tomography demonstrating subcutaneous emphysema in the cervical fascia

**Figure 3:**
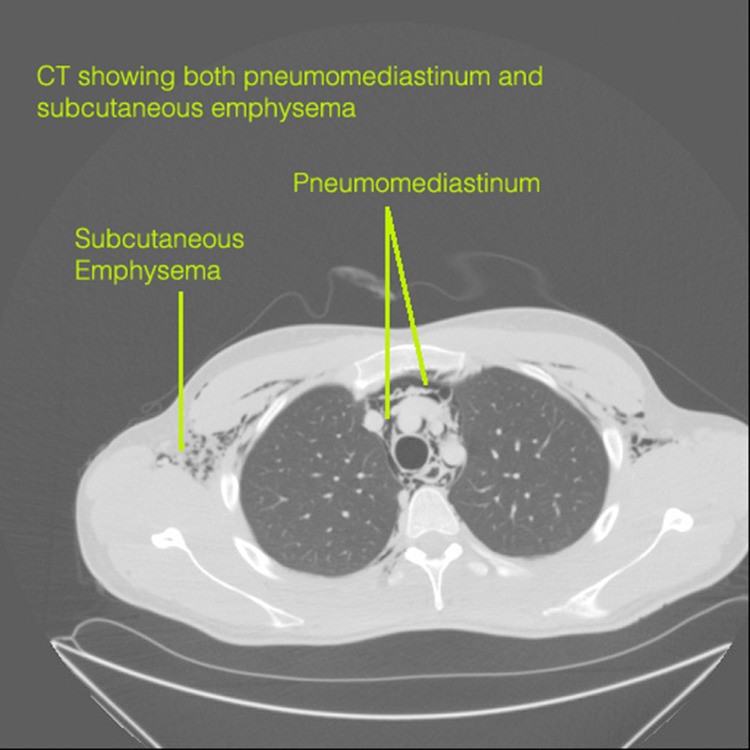
Computed tomography demonstrating pneumomediastinum and subcutaneous emphysema

The diagnosis of spontaneous pneumomediastinum is based on the clinical triad of chest pain, dyspnoea and subcutaneous emphysema.^2,8^ These factors were the most common findings in Abolnik *et al’*s study of 25 cases of spontaneous pneumomediastinum.^9^ Chest pain was found in 88%, dyspnoea in 60% and subcutaneous emphysema in 60%. Infrequently associated with spontaneous pneumomediastinum are rhinolalia, dysphagia, anxiety and sore throat.^2^ In our case, rhinolalia and pain were the most obvious findings. Rhinolalia is described rarely in medical literature. However, Hoover *et al* present it as a thickened, high pitched, hyponasal voice occurring due to retropharyngeal collection of air.^10^

## Conclusions

The complications associated with pneumomediastinum include tension pneumomediastinum and unilateral, bilateral or tension pneumothorax. Fortunately, these complications are rare and the condition will usually have a benign, uncomplicated cause.^11^ Treatment can be based on simple measures such as patient reassurance, rest and analgesia. In cases of persistent subcutaneous emphysema, inhalation of high concentration oxygen has been shown to be beneficial.^12^
